# Comparative pan-genomic analyses of *Orientia tsutsugamushi* reveal an exceptional model of bacterial evolution driving genomic diversity

**DOI:** 10.1099/mgen.0.000199

**Published:** 2018-07-23

**Authors:** Amy Fleshman, Kristin Mullins, Jason Sahl, Crystal Hepp, Nathan Nieto, Kristin Wiggins, Heidie Hornstra, Daryl Kelly, Teik-Chye Chan, Rattanaphone Phetsouvanh, Sabine Dittrich, Phonepasith Panyanivong, Daniel Paris, Paul Newton, Allen Richards, Talima Pearson

**Affiliations:** ^1^​ Northern Arizona University, Flagstaff, AZ, USA; ^2^​ Naval Medical Research Center, Silver Spring, MD, USA; ^3^​ The Ohio State University, Columbus, OH, USA; ^4^​ Lao-Oxford-Mahosot Hospital-Wellcome Trust, Research Unit, Mahosot Hospital, Vientiane, Vientiane, Lao People's Democratic Republic; ^5^​ University of Oxford, Centre for Tropical Medicine and Global Health, Oxford, UK; ^6^​ Lao-Oxford-Mahosot Hospital-Wellcome Trust Research Unit, Mahosot Hospital, Vientiane, Lao People's Democratic Republic; ^7^​ Foundation of Innovative New Diagnostics, Geneva, Switzerland; ^8^​ Mahidol-Oxford Tropical Medicine Research Unit, Bangkok, Thailand; ^9^​ Swiss Tropical and Public Health Institute, Basel, Switzerland; ^10^​ University of Basel, Basel, Switzerland; ^11^​ Uniformed Services University of the Health Sciences, Bethesda, MD, USA

**Keywords:** adaptive evolution, gene divergence, genome adaptation, lateral gene transfer, pan-genome, scrub typhus

## Abstract

*Orientia tsutsugamushi*, formerly *Rickettsia tsutsugamushi*, is an obligate intracellular pathogen that causes scrub typhus, an underdiagnosed acute febrile disease with high morbidity. Scrub typhus is transmitted by the larval stage (chigger) of *Leptotrombidium* mites and is irregularly distributed across endemic regions of Asia, Australia and islands of the western Pacific Ocean. Previous work to understand population genetics in *O. tsutsugamushi* has been based on sub-genomic sampling methods and whole-genome characterization of two genomes. In this study, we compared 40 genomes from geographically dispersed areas and confirmed patterns of extensive homologous recombination likely driven by transposons, conjugative elements and repetitive sequences. High rates of lateral gene transfer (LGT) among *O. tsutsugamushi* genomes appear to have effectively eliminated a detectable clonal frame, but not our ability to infer evolutionary relationships and phylogeographical clustering. Pan-genomic comparisons using 31 082 high-quality bacterial genomes from 253 species suggests that genomic duplication in *O. tsutsugamushi* is almost unparalleled. Unlike other highly recombinant species where the uptake of exogenous DNA largely drives genomic diversity, the pan-genome of *O. tsutsugamushi* is driven by duplication and divergence. Extensive gene innovation by duplication is most commonly attributed to plants and animals and, in contrast with LGT, is thought to be only a minor evolutionary mechanism for bacteria. The near unprecedented evolutionary characteristics of *O. tsutsugamushi*, coupled with extensive intra-specific LGT, expand our present understanding of rapid bacterial evolutionary adaptive mechanisms.

## Data Summary

1. All raw sequence data were deposited in the National Center for Biotechnology Information database under BioProject PRJNA316643: https://www.ncbi.nlm.nih.gov/bioproject/?term=PRJNA316643.

2. Draft genome assemblies are available at: https://github.com/jasonsahl/OT_genomics.

3. All other data generated and analysed for this study are included as follows: Additional files 1, 4 and 12 are included as supplementary data (available with the online version of this article), while Additional files 2, 3, 5, 6, 7, 8, 9, 10, 11 and 13 have been deposited in Figshare: https://doi.org/10.6084/m9.figshare.5995456.v1.

**Impact Statement**

*Orientia tsutsugamushi* genomes reveal great phylogenetic and genomic diversity. By comparing 40 whole-genome sequences from diverse geographical locations, we demonstrate that the evolutionary history of *O. tsutsugamushi* includes high levels of homologous recombination in the form of lateral gene transfer (LGT) within this species. LGT is so widespread in the genome that it has degraded most, if not all, of the clonal frame (portions of the genome that have been strictly vertically inherited). While pan-genome diversity of other bacteria is often driven by incorporating genetic material from outside taxa, the pan-genome of *O. tsutsugamushi* is driven primarily by gene duplication and divergence, a characteristic usually attributed to eukaryotes. Pan-genomic comparisons to 253 bacterial species demonstrate that such extensive duplication and divergence is extremely rare among bacteria. Proliferative duplication and extensive intra-specific LGT in *O. tsutsugamushi* may be fundamental to the evolutionary adaptation to diverse hosts, vectors and habitats.

## Introduction

The obligate intracellular bacterial pathogen and causal agent of scrub typhus, *Orientia tsutsugamushi*, causes an estimated one million cases of scrub typhus annually across regions containing over one billion people [1]. Scrub typhus is primarily found in the Asia-Pacific region [2], but our knowledge of the distribution has expanded this extent to regions in Africa, Chile and the United Arab Emirates in recent years [3–5]. The clinical presentation of scrub typhus is highly variable across individuals and regions, and includes fever, lymphadenopathy, myalgia, pneumonitis and multiple organ failure, accompanied by high morbidity and death. Humans, birds, rodents and small mammals can be infected with *O. tsutsugamushi* when fed upon by infected larval (chigger) trombiculid (*Leptotrombidium* spp.) mites. Within the mite population, *O. tsutsugamushi* is transmitted transovarially or transstadially [6–8]. Multiple genotypes may occur within a single mite [9].

The diverse ecology, distribution, clinical presentation and inability to design a suitable vaccine for scrub typhus is reflected in the genomic evolution of *O. tsutsugamushi*, which facilitates rapid adaptation and host shifting through extensive lateral gene transfer (LGT), amplification, rearrangement and decay [10, 11]. LGT in other bacteria is the main system for securing exogenous gene content to facilitate adaptation and causes isolates to differ in their genomic content. Gene duplication represents another mechanism for generating diversity and is thought to be the most important adaptive evolutionary mechanism in eukaryotes [12]. In contrast, prokaryotic genome evolution is dominated by deletion and acquisition through LGT [13, 14], although extensive duplication has been documented in some species [15, 16]. Duplication is an important mechanism for adaptation to the environment; however, this form of evolution is slower than LGT [14, 15]. Alterations in paralogues may escape strict stabilizing selection and ultimately lead to neofunctionalization. Alternatively, duplication of a pleiotropic gene may lead to the partitioning of function among gene copies, leading to adaptive specialization of functions and limiting the impact of subsequent divergence to a single function. For *O. tsutsugamushi*, the total length of duplicated genes accounts for >37 % of the Boryong genome [11] and 46.5 % of the Ikeda genome [10]. The proportion of duplicated genes is larger than any other bacterial species sequenced [11, 17]. No plasmids or prophages have been found in these genomes, but high densities of repeated integrative and conjugative elements, as well as transposable elements, provide the necessary genomic foundation to facilitate additional gene amplification and LGT [10, 11]. Repetitive sequences serve as recombination hotspots by promoting intra-chromosomal rearrangement via homologous and non-homologous recombination and LGT [18–20]. Duplicated sequences are also highly susceptible to gene decay [18, 19, 21], generating flux within the *O. tsutsugamushi* genome through duplication-divergence events. The deletion of genes associated with DNA recombination and repair systems in *Rickettsia* species may facilitate the maintenance of duplicated genes and their derivatives [10].

With only two publicly available whole-genome sequences, previous studies have used sub-genomic methods to characterize *O. tsutsugamushi* populations [2, 5, 22, 23]. Although analyses using single genes can potentially model evolution within certain populations, they cannot infer evolution at the genome level for highly recombinant species. Previous *O. tsutsugamushi* analyses that have used multi-locus sequence type (MLST) methods demonstrate that populations are highly genetically diverse [24–26]. However, as with single genes, MLST genes represent only a small portion of the genome. Phylogenetic analyses of MLST genes, therefore, produce a much different phylogeny for some species than one using the entire genome [27]. High levels of recombination due to LGT result in a chimeric genome composed of horizontally acquired regions with different evolutionary histories. However, even in species with high levels of LGT, some genomic regions, known as the clonal frame [28], are likely to be strictly vertically inherited even over long periods of evolutionary time due to stabilizing selective pressures. Previous studies using MLST have shown high levels of recombination in *O. tsutsugamushi* through the ratio of recombination to mutation (r/m) in single locus variants [25]. For *O. tsutsugamushi*, the ratio at which recombination gives rise to new MLST alleles compared to mutation has been measured at approximately 10 times [25] and 17 times [29]. While this rate is far less than the rate in the human pathogen and soil saprophyte *Burkholderia pseudomallei* [30], it is greater than or comparable to other human pathogens [25, 30] where LGT has played an important role in shaping the evolution of the species. For many intracellular pathogens, there is little opportunity for interaction and LGT among conspecific strains. However, multiple *O. tsutsugamushi* strains have been discovered in ~25 % of human infections [25] by MLST and co-infections of laboratory mites and other human clinical cases have also been observed [9, 31].

In this study, we used the whole-genome sequences of 40 *O. tsutsugamushi* isolates from South-East Asia, islands of the western Pacific, Australia and Pakistan to investigate evolution and diversity at a genomic and population level of resolution that has not been previously described for *O. tsutsugamushi*. We examine the distribution of LGT across the phylogeny and the genome to determine possible sources of LGT and chromosomal regions that may be highly conserved and not subject to the potentially deleterious effects of recombination. Lastly, we investigate the roles of LGT and genetic duplication in adding to the pan-genome diversity of the species. Our results provide insight into a mechanism of microbial pan-genome adaptive evolution, the extent of which has not been previously documented for any other bacterial species. Through routine analyses of a bacterial genome with high levels of LGT and repetitive sequences, we also highlight the limitations and potential of current tools to address comparative genomics analyses.

## Methods

### DNA isolation and extraction

DNA preparations of *O. tsutsugamushi* isolates (*n*=33) were received from the Naval Medical Research Center (NMRC) in Silver Spring, MD, USA, and the Lao-Oxford-Mahosot Hospital-Wellcome Trust Research Unit (LOMWRU) in Vientiane, Lao PDR (Laos). Isolates originated from a variety of geographical locations from different hosts and times. Epidemiological information for each isolate can be found in Additional file 1. Lao isolates were isolated from patient buffy coat and grown in Vero and L929 cells at LOMWRU as described by Phetsouvanh *et al.* [29]. NMRC isolates were grown from frozen seed stocks and inoculated directly into irradiated L929 cells. DNA extraction procedures are detailed in Additional file 1.

### Genome sequencing, assembly and annotation

Library preparations were performed using the same protocol as described in Keim *et al*. [32], with the following modifications: approximately 1 µg DNA per sample was fragmented using a Q800R2 sonicator (QSonica) with the following parameters: 3 min sonication with 15 s pulse on, 15 s pulse off, and 20 % amplitude [32]. For samples that were dual-indexed, the second 8 bp index oligonucleotides purchased from IDT based on the work of Stone *et al*. [33] were used in addition to reduce optical sequencing contamination [33]. All samples were sequenced on an Illumina HiSeq 2000 using the 200-cycle TruSeq SBS kit v3-HS (Illumina). Sequencing primers were added to the HiSeq kit as described by Kozarewa and Turner [34]. Sequenced genomes were assembled with SPAdes v. 3.6.0 [35] in conjunction with a high throughput assembly pipeline (https://github.com/jasonsahl/UGAP). As isolates were cultured in cells derived from *Mus musculus* (L929) and *Chlorocebus sabaeus* (Vero), reads that aligned to genome assemblies of these species were removed prior to genome assembly. As part of the ugap pipeline, reads were mapped to each contig in each genome assembly and removed if per contig depth of coverage was anomalously low compared to the rest of the assembly based on read mapping with genomeCoverageBed, part of bedtools [36, 37]. To identify cross-species contamination, we used blastn [38, 39] to align the first 200 bases of each contig in each assembly against the GenBank nt database. Contigs with no significant blast hits were examined further to examine the quality and G+C content of each contig. Contigs aligning to host sequences or known contaminants, including from other organisms multiplexed in the same run, were removed from assemblies. Five additional draft assemblies were retrieved from GenBank in fasta format [40]. All raw sequence data were submitted to the National Center for Biotechnology Information (NCBI) under BioProject PRJNA316643 and SRA accession number SRP075135. Additional identifiers for each isolate are located in Additional file 1.

### Whole-genome single nucleotide polymorphism (SNP) discovery and phylogeny

All SNPs (Additional file 2) were identified with the nasp pipeline [41]. nasp was used to align raw reads with bwa-mem [42] and external genome assemblies with NUCmer [43] to the finished reference (Boryong) genome (NC_009488.1). Only SNP loci found in all genomes were included. Regions duplicated in the Boryong genome were identified by a NUCmer self-alignment (1 177 863 bp; Additional file 3) and filtered from all subsequent SNP analyses. For sequence reads, SNPs were called using the UnifiedGenotyper method in gatk [44] and SNPs were filtered based on 3× read depth and 90 % allele frequency. SNPs were retained if they had a valid call in 100 % of all genomes.

*Rickettsia bellii* was identified as a close neighbour to *O. tsutsugamushi* [45] and, thus, an appropriate outgroup for rooting the *O. tsutsugamushi* phylogeny (Additional file 4). To identify the most basal *O. tsutsugamushi* genome, we inferred maximum-parsimony (MP) and neighbour-joining (NJ) trees with paup [46] and maximum-likelihood (ML) trees with iq-tree v1.6 [47] on 59 363 whole-genome SNPs and concatenated ribosomal proteins (*n*=41) rooted with *R. bellii*. To produce the *O. tsutsugamushi* phylogeny, we used SNPs from loci present in all *O. tsutsugamushi* genomes to generate MP and NJ trees with 1000 bootstrap replicates using paup [46] and ML trees using iq-tree v1.6 [47] with automatic selection of evolutionary model.

In an effort to better understand the impacts of recombination on phylogeny, we additionally employed beast v1.8.0 [48] to generate a distribution of trees. LGT that occurred frequently between the same lineages can be expected to leave a phylogenetic footprint that will appear in the distribution of trees. Details on beast runs and methodologies can be found in Additional file 1. To visualize the distribution of trees resulting from the beast analysis, DensiTree [49] was employed, where the two consensus trees representing 95.37 % of the 1800 randomly sampled trees (from the 400 000 000 trees generated) were overlaid (Additional file 4).

From the *O. tsutsugamushi* parsimony tree, we identified homoplastic SNPs in paup [consistency index (CI) <1] [46]. To help understand the extent and distribution of recombination within the *O. tsutsugamushi* phylogeny, parsimony trees were also drawn using only 27 027 homoplastic SNPs identified from the previous parsimony analysis.

### SNP density (SD) and homoplasy density (HD) across the *O. tsutsugamushi* genome

SD and HD [50] values were computed to quantify and visualize spatial patterns of recombination across *O. tsutsugamushi* genomes. Homoplasy and regions with increased densities of SNPs can also be due to recurrent point mutations and regions under heavy selection or drift; however, high levels of homoplasy do not occur in clonally propagated genomic regions [51–58] and heterogeneity in point mutation rates is low [59]. As such, genomic scans for homoplasy and elevated SDs can, respectively, indicate recombination within a group and from exterior sources [57, 58, 60] and form the foundation for sophisticated recombination detection software [61–63]. The SD was computed by counting the number of parsimony informative (PI) SNPs (identified by paup [46]) across 1 kb non-overlapping windows in the Boryong genome. The HD was computed by identifying PI SNPs with a retention index (RI) below 1.0 in the same 1 kb non-overlapping window. The RI was calculated using paup v4 [46]. HD and SD were mapped along the circular Boryong genome using Circos [64].

### Search for evidence of recombination and a clonal frame in *O. tsutsugamushi*

To further explore the extent of recombination throughout the *O. tsutsugamushi* genome, we used PhiPack [65, 66] to identify incongruent SNPs in an alignment of monomorphic and polymorphic sites in the nasp SNP matrix. PhiPack computed a refined incompatibility score (*P* value) for each designated window of 25 bp, scanning window of 250 bp and step size of 25 bp [65, 66] across the alignment. In order to test whether regions identified as non-recombinant were indeed clonal and, thus, represented a clonal frame for *O. tsutsugamushi*, we extracted each non-recombinant region from genome assemblies, as identified by PhiPack, aligned the sequences with muscle [67], inferred an MP tree with Phangorn [68] and calculated the RI for each tree. We assumed that an RI of <1.0 for any tree may indicate recombination in that region. Regions that produced trees with RI values of 1.0 (*n*=491) remained candidates for a clonal frame and were further tested by concatenating positions from these regions and drawing an MP tree with Phangorn. Putative clonal regions would be expected to maintain low homoplasy even when concatenated and may not present in a contiguous region of the genome.

ClonalFrameML [63] and Gubbins [61] were also used to identify potential recombination across *O. tsutsugamushi* genomes. We used the multiple sequence alignment (MSA) and tree produced by Gubbins to calculate CI and RI values and confirm the presence of recombinant and non-recombinant sites throughout *O. tsutsugamushi* genomes. For ClonalFrameML, we removed putative recombinant regions and clonal regions within duplicated regions before searching for SNPs, drawing an MP tree, and calculating CI and RI values. Default parameters were used for ClonalFrameML and Gubbins.

### Pan-genome, core genome, unique genes and duplicate region analyses

We used the large-scale blast score ratio (LS-BSR) pipeline [69] to determine conservation of genes and quantify the extent of gene duplications across *O. tsutsugamushi* genomes. Coding sequences (CDSs) with a BSR ≥0.8 across all genomes were considered to be conserved, absent with a BSR <0.4, and divergent with a BSR between 0.8 and 0.4 [70]. LS-BSR was also used to calculate the size and content of the *O. tsutsugamushi* pan-genome. A BSR matrix was created (Additional file 5) that included the BSR value for each CDS across each query genome, a list of CDSs duplicated in at least one genome and a multi-fasta of all unique CDSs (those present in only a single genome) (Additional file 6). To understand the annotation of unique regions, all unique CDSs were aligned against the GenBank nt database using blastn [38] and annotation was transferred for significant alignments (Additional file 7).

Additionally, LS-BSR was used to identify the core genome using *O. tsutsugamushi* assemblies, which produced a small core genome size, likely due to highly fragmented assemblies caused by un-resolvable and collapsed repeats. To address this limitation, we calculated the size of the core genome using raw sequence data instead of assemblies. First, we used LS-BSR to identify the core genome (all CDSs where BSR ≥0.8) when just analysing the two finished *O. tsutsugamushi* genomes (core genome size=960 CDSs). For consistency, we wanted to run the analysis with the same datatypes, in this case raw read data. Therefore, simulated paired-end Illumina reads for each external genome in the analysis (*n*=5) were produced with 100 bp read length, 50× fold coverage, a mean size of 300 bp, a standard deviation of 50 bp fragments and Illumina HiSeq 2500 error profiles using ART-ChocolateCherryCake [71]. Simulated reads and raw sequence data generated in this study were aligned against the finished genome core with bwa-mem and the per base coverage was calculated with genomeCoverageBed [36, 37]. The breadth of coverage, or percentage of the reference gene covered by three or more reads, was then calculated. CDSs with a breadth of coverage of >80 % across all genomes were considered to be core genes. Core positions from the reads in Boryong were mapped onto a circular genome map with Circos [64] and can be found in Additional file 8 (Fig. 2).

We also used LS-BSR to identify the size of the pan-genome at different levels of sequence identity during the clustering stage, using usearch [72]. The BSR_to_gene_accumulation_scatter.py script was used to subsample the genomes and compute the pan-genome, using 10 replicates; core and pan-genome plots were generated using the methods described by Sahl *et al.* [69].

### Comparative pan-genomics

To understand how the size of the pan-genome changed by clustering at lower identities, all bacterial genomes were downloaded from GenBank on October 25th 2017. Genomes were filtered out if they contained more than 10 ambiguous characters, or contained an anomalous number of contigs, genome assembly size or pairwise mash [73] distance; anomalous values were identified by two standard deviations away from the median value. Only genomes with more than 10 high-quality (as defined by Chain *et al*. [74]) genomes/species were included. A final list of all genomes used in this study (*n*=31 082) are in Additional file 9. usearch v9.0.2132 [72] and vsearch v2.0.4 [75] were run on all coding regions in each species at varied identity thresholds and the number of unique clusters was quantified and plotted.

## Results

In order to identify phylogenetic and phylogeographical signals, understand mechanisms of evolution, and explore the diversity of the pan-genome of *O. tsutsugamushi*, we sequenced and analysed 33 genomes from isolates collected in South-East Asia. Additionally, we analysed the two published complete genomes, Boryong and Ikeda, and five draft assemblies. We combined these results with pan-genomics analysis of all bacterial genomes in GenBank in order to correlate genomics with pathogen ecology.

### Genome assembly

Genome assembly methods using short reads are not well suited for genomes with high frequencies of repeated elements [76]. Because repeat sequences comprise ~40–50 % of the *O. tsutsugamushi* genome [10, 11], our genome assemblies were small and highly fragmented, most likely due to repeat collapse [17]. To demonstrate the effect of un-resolvable repeats on the quality of the genome assembly, we simulated Illumina reads from the completed Boryong genome and assembled them with the same pipeline that was used for real sequence data. The results demonstrated that the size of the genome assembled from simulated reads was only 1 590 159 bp in length, which is 25 % shorter than the completed genome assembly size (2 127 051 bp). The small, fragmented assemblies derived from short reads submitted to GenBank (Additional file 1) produced in this study (assemblies available at https://github.com/jasonsahl/OT_genomics.git) were not used for SNP discovery, recombination analyses and pan-genome analyses and highlights the limitations of using highly repetitive genome assemblies from short-read sequence technologies.

### Whole-genome SNP discovery and phylogenomic analyses

**Fig. 1.**
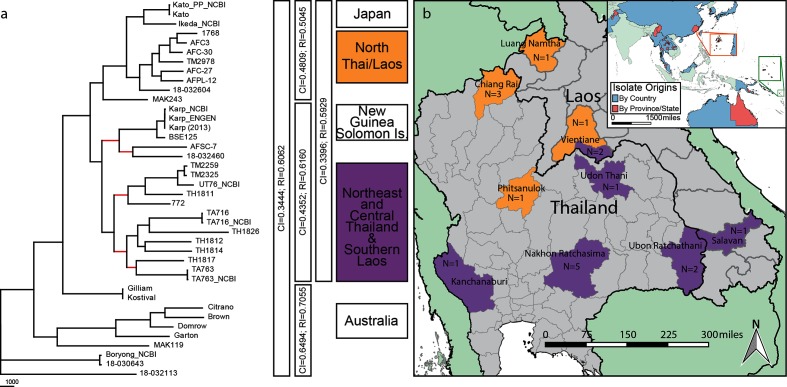
Evolutionary relationships and geographical locations of samples. (a) MP tree inferred from 59 363 SNPs among 40 whole-genome sequences. Branches with bootstrap support of <70 % are shown in red. The total tree length is 143 686. The CI (excluding parsimony uninformative characters) and RI values for different sets of genomes are indicated to the right of the tree. Geographical region of samples corresponding to different clades are also shown to the right of the tree. (b) Map of South-East Asia (inset) and Thailand/Laos showing sample origins and coloured according to clade designation.

Rooting a phylogeny is critical for understanding evolutionary relationships among taxa, evolutionary rates, geographical dispersal, adaptations and directionality of evolution [55]. We used standard outgroup rooting to identify the root for *O. tsutsugamushi*. SNPs were identified for all 40 *O. tsutsugamushi* genomes compared to the reference (Boryong) and 2 genomes of *R. bellii,* a close relative based on comparative 16S rRNA sequence analysis [45]. Among the *O. tsutsugamushi* genomes, we identified 59 363 SNP loci present in all genomes, although only 337 of these loci were present in the two *R. bellii* genomes. Analyses of these 337 positions produced four MP phylogenies with low consistency and RI values (CI=0.44; RI=0.61) and a bootstrap consensus tree with a basal polytomy for the *O. tsutsugamushi* genomes when nodes with <50 % bootstrap support were collapsed (Additional file 4). Conversely, a NJ bootstrap consensus tree and an ML bootstrap consensus tree showed single branches (72 and 69 % support, respectively) with single genomes (18-032113 and TH1826, respectively) as the most basal *O. tsutsugamushi* genomes (Additional file 4). Additional analyses using concatenated ribosomal protein sequences (with 1564 variable characters of which 1454 were PI) all identified 18-032113 as the most basal taxa, supported by 98, 84 and 71 % of 1000 NJ, ML and MP (respectively) bootstrap iterations (Additional file 4). Given these results, 18-032113 was used to root all phylogenies involving only *O. tsutsugamushi* genomes. MP analyses of the 40 *O. tsutsugamushi* genomes inferred a single most parsimonious tree using the 59 363 SNPs from the core genome (Fig. 1). The CI (excluding parsimony uninformative characters) was 0.34 and the RI was 0.61, indicating high levels of homoplasy. Nonetheless, high bootstrap values were obtained for most nodes on the tree (Additional file 4), providing confidence for many parts of the phylogeny. Furthermore, ML methods produced a tree with 96.9 % topological similarity to the MP tree, assessed using compare2trees [77], while Bayesian and NJ phylogenetic methods produced trees with topological scores >90 % compared to the MP tree.

Comparing the alternate topologies created within and across phylogenetic methods can show the regions of topological uncertainty and, thus, provide insights into patterns of lateral gene exchange as well as differences in phylogenetic algorithms. Most phylogenetic groupings of *O. tsutsugamushi* are conserved across trees, suggesting that LGT most frequently occurred within, rather than across, groups. Conversely, more frequent LGT across lineages may explain the phylogenetic inconsistencies for the few genomes whose positions vary across trees. Phylogenetic analyses of only homoplastic SNPs can also provide important clues about gene sharing across lineages. When LGT disproportionately involves certain lineages (highways of gene sharing), the phylogenetic position of these lineages can be drastically altered [78]. Conversely, LGT that occurs randomly across lineages will not destroy vertical inheritance patterns [30, 79] even if the frequency increases with the relatedness of the genomes. Indeed, our analysis of only homoplastic SNPs resulted in a topology highly similar to MP (91 % tree congruence), NJ (94.2 % congruence) and ML trees (88.7 % congruence) constructed using all SNPs (Additional file 4). Together, these analyses provide support for a mostly random model of LGT that primarily occurs within groups.

All phylogenetic trees show a paraphyletic basal group and two monophyletic clades (Fig. 1, Additional file 4). While the topologies within some of these groups vary slightly across trees, their membership does not, and phylogeographical structuring is evident. Our sampling of isolates outside the Thailand/Laos region is sparse, but does suggest phylogeographical structuring (Fig. 1). The basal paraphyletic group contains a monophyletic clade containing only Australian isolates. The isolates from Japan (Kato and Ikeda) group together, and the isolates from Papua New Guinea, Solomon Islands and Malaysia (Karp, bse125 and 18032460) also group together. A Thai isolate (afsc7) is placed within this group in the MP tree (Fig. 1) and the major beast tree (Additional file 4), but not in any of the bootstrap consensus trees (Additional file 4). Two monophyletic clades exclusively contain all of the genomes from Thailand and Laos. One clade contains isolates from the northern part of Thailand/Laos, while the other contains isolates from the southern part of Laos and north-eastern and western Thailand (Fig. 1). This phylogeographical structuring is not absolute as one isolate from Vientiane (TM2978) falls in the northern clade, while two from Vientiane (TM2259 and TM2325) are placed in the southern clade. This is consistent with MLST analyses of a larger number of isolates that showed isolates from Vientiane associated with different regions, possibly reflecting the importance of Vientiane as a hub for human transit [29]. While most isolates from similar geographical regions tend to group together, the Boryong (Korea) and 18032643 (China) isolates differ only by 207 SNPs, despite being collected ~1700 km apart. Likewise, the isolates Gilliam and Kostival differ by four SNPs despite being collected in 1944 and 1943 from Burma and Papua New Guinea, respectively. Given the extensive genetic diversity found even at a local spatial scale, it is likely that these two samples have been mixed at some point since 1944. Sequencing the 56 kDa genes from our original Kostival stock received in 1992, in addition to stocks dating back to 1975, yielded sequence matches to the whole genome (data not shown). If the Kostival strain was contaminated, it was done so before 1975. Reads from these same genes in our Gilliam genome matched those submitted to NCBI by other labs, again suggesting that if the Gilliam strain was mixed, it was done so before this strain was disseminated to other labs.

### Recombination analyses

**Fig. 2.**
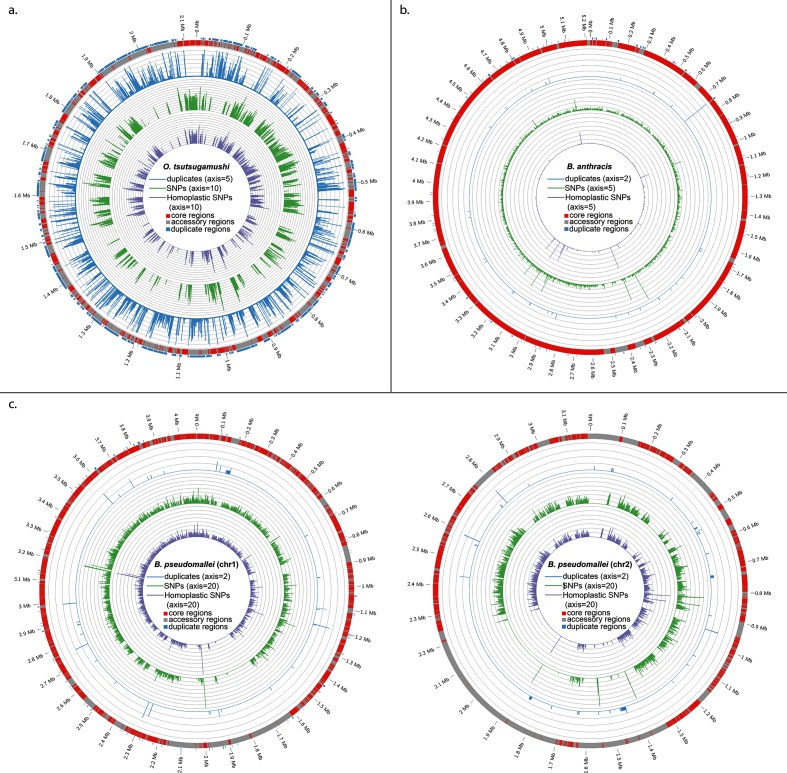
Pan-genome characteristics of *O. tsutsugamushi*. (a) Spatial designation of pan-genome characteristics against the Boryong genome. Outer to inner rings designate the location of the core regions of the genome, repeat location and frequency, SNP frequency (core regions only), and homoplasy frequency (core regions only). Unit size for each marker on the axis is provided in the central key. Spatial designations of these same characteristics for *Bacillus anthracis* (b) and *Burkholderia pseudomallei* (c).

Regions transferred from phylogenetically distant genomes (from an outgroup) tend to include more SNPs, while gene exchange between closely related taxa (within the ‘ingroup’ or group of interest) are typified by homoplasy. Mapping SD and HD of these SNPs can provide insights into the genomic locations of recombination events in bacteria [60]; however, low levels of homoplasy and regions with slightly elevated SDs can also be caused by point mutations and selective pressure. SD and HD are distributed across the Boryong strain shown in the circular map (Fig. 2a). The majority of the regions with high SD also have high HD, suggesting that the source of most transferred DNA is within the sampled phylogeny. For contrast, we also show the distribution of SD and HD across *Bacillus anthracis* (Fig. 2b), a clonal species [54], and *Burkholderia pseudomallei* (Fig. 2c), an organism associated with high levels of LGT and recombination [30]. The results demonstrate that the amount of homoplasy far exceeds what can be expected in clonal bacterial pathogens.

Considerations of SD and homoplasy also form the foundation of ClonalFrameML [63], an algorithm used to assess rates and genomic locations of recombination across large numbers of bacterial genomes. Using the 40 *O. tsutsugamushi* genomes, ClonalFrameML estimated *R*/*θ*=1.057, *δ*=190 bp and *ν*=.0317, suggesting that recombination events involve short tracts of DNA (190 bp on average), occur 1.06 times as frequently as mutation, but typically introduce 6× more substitutions than mutation. This estimated relative effect of recombination and mutation is less than the ~10× and ~17× previously estimated using MLST loci [25, 29]. ClonalFrameML is designed specifically to detect recombination when sources are external to the sampled population, but consistently underestimates *R*/*θ* by an order of magnitude when sources are primarily from within the sampled species [63]. This underestimation is most severe when the recombined regions are small (as with *O. tsutsugamushi*), and may be as much as >1.5 orders of magnitude [63]. SD and HD patterns, as well as pan-genome analyses (see below), suggest that sources of transferred DNA are primarily from within the represented phylogeny. Therefore, accounting for an underestimation of *R*/*θ* by 1.5 orders of magnitude suggests that recombination events may have occurred ~33 times more frequently than mutation, and introduced ~201 times more substitutions than mutations. Such a high relative rate has not been previously documented.

The ‘Profile’ script implemented in PhiPack [65, 66] also indicated the presence of recombination throughout the genome alignment by conducting 84 380 tests (based on the size of a sliding window and step sizes across the genome): 17 954 (21 %) indicated regions of the genome alignment that are likely recombinant where *P*≤0.05 and 66 426 (79 %) corresponded to regions that are likely non-recombinant (*P*>0.05) or do not contain sufficient variation to identify recombination. Out of 40 699 putatively non-recombinant regions that contained sufficient polymorphisms, we identified a subset of 491 regions that presented the strongest evidence of clonality (RI=1 across all genomes) for further investigation. To investigate the possibility that these regions represent the clonal frame of *O. tsutsugamushi*, we concatenated the sequences of these 491 regions into a nearly 123 kb alignment and inferred an MP tree. Though we expected the tree to have an RI close to 1, the observed RI was 0.54, indicating that at least some of these regions have not been strictly vertically inherited and have been subject to LGT. To determine if any of these regions are phylogenetically consistent with each other, we looked at their positions in the Boryong genome and identified a contiguous 725 bp region with an RI of 0.9, suggesting that this region, associated with a GTPase (locus tag=OTT_0266), demonstrates a potentially clonal pattern of inheritance. Assessing putatively clonal regions by determining the RI of concatenated regions is not reliant on congruence with the overall phylogenetic tree. However, this region produced a tree (Additional file 4) with a low topological score, 46.3 %, compared to the overall MP tree, assessed using compare2trees.

We also used Gubbins and ClonalFrameML to identify recombinant regions. SNPs associated with recombined regions can be removed, leaving a putative clonal frame. Gubbins, using 59 363 SNP loci from 40 genomes, inferred 499 recombination events and produced a tree excluding recombinant loci with a CI of 0.48 and an RI of 0.62. The RI of the putatively non-recombinant tree is the same as the RI of the parsimony tree inferred from the whole dataset. From the 40 genomes, ClonalFrameML inferred 19 425 importation events and 158 putatively clonal regions that are not in duplicated regions (Additional file 10). The mean size of these putatively clonal regions is 131 bp. A tree produced from SNPs in these putatively non-recombinant and non-duplicated regions yielded an RI of 0.77 and CI of 0.65. Trees inferred from the putative clonal regions identified by Gubbins and ClonalFrameML have low CI and RI values, indicating that at least some of these regions must be recombinant. The trees derived from the Gubbins and ClonalFrameML analyses were highly similar (98.2 and 86.2 %, respectively) to the MP tree constructed using the full data set (Additional file 4).

### Pan-genome, core genome and duplicate regions analyses

**Fig. 3.**
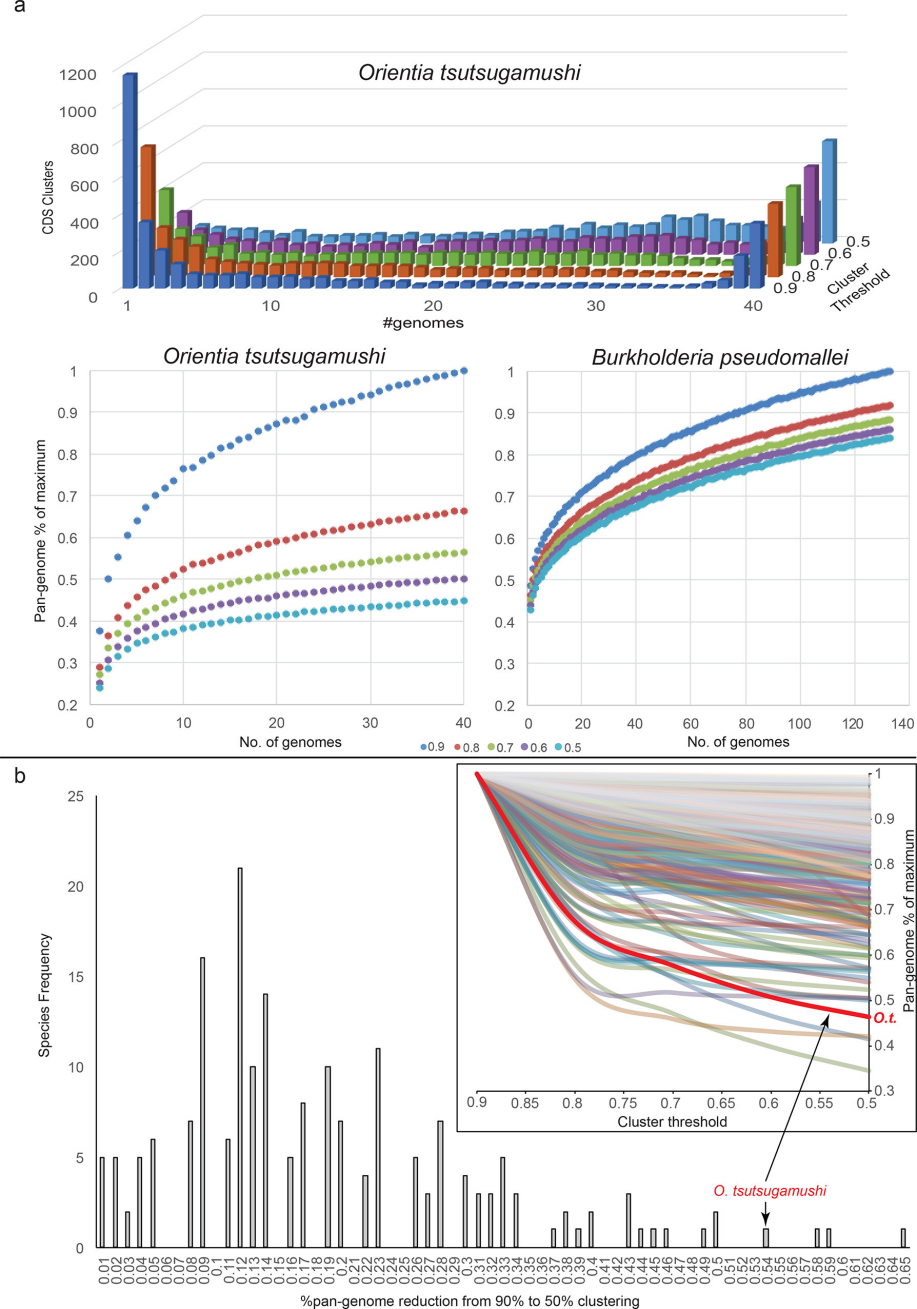
Comparative pan-genomic analysis. Pan-genome analysis of *O. tsutsugamushi* and (a) *Burkholderia pseudomallei* and (b) other bacterial species using a range of thresholds to identify homologous genes. As the per cent identity threshold is decreased, increasingly divergent genes cluster together, resulting in a larger number of genes shared by most genomes and a smaller pan-genome size. For *O. tsutsugamushi*, the reduction in pan-genome size using lower identity thresholds is striking and greater than *Burkholderia pseudomallei* (a) and all but 3 of 253 other bacterial pan-genomes (b).

Due to fragmented assemblies, LS-BSR only identified a total of 362 conserved genes (BSR ≥0.8 in all genomes). From the sequencing read data, the number of regions that had a breadth of coverage of >70 % (at 3× minimum depth) were identified and resulted in 687 core genes (Additional file 11). A total of 79 unique genes (those with a BSR <0.4 in all but one genome) across all strains were also identified; 2 of these unique genes were found in two genomes each, but represent re-sequencing efforts of the same strain (TA716). The majority of unique genes showed a close homologue to other *O. tsutsugamushi* genomes that were annotated as outer-membrane proteins, conjugative genes or mobile elements. Furthermore, seven of these genes that are unique to one genome have multiple copies. Using different per cent identity thresholds significantly impacted the number of identified ‘unique’ gene clusters, resulting in drastically different estimations of pan-genome size (Fig. 3a). For higher levels of gene identity, more non-core gene clusters were found and a plot of the total number of clusters against the total number of genomes demonstrated logarithmic growth (Fig. 3a), suggesting an open pan-genome. In the same analysis using lower levels of gene identity, we found fewer strain-specific gene clusters (Fig. 3a, Additional File 7), providing additional evidence that most unique genes and many other genes found in only a small number of genomes are actually divergent paralogues. Using lower levels of gene identity causes divergent paralogues to cluster together and, thus, not contribute to an increase in the pan-genome. Therefore, at low gene identity levels, the pan-genome size rapidly plateaus, indicating that the pan-genome appears to be closed and fully sampled with only a small number of genomes.

To explore the reduction in the core genome due to relaxed clustering thresholds, we extracted one of the unique proteins out of Ikeda and Boryong using blast. The multiple sequence alignment (Additional file 12) demonstrates that although homology exists between the unique protein and proteins in Boryong and Ikeda, clear differences exist that would make the proteins appear different at higher cluster thresholds, but similar at lower thresholds. These regions increase the pan-genome at higher cluster thresholds, but clearly share homology with other proteins in other *O. tsutsugamushi* genomes.

To determine the frequency of this phenomenon of collapsed pan-genomes with decreased clustering thresholds among other species, we downloaded all bacterial genomes from GenBank with more than 10 high-quality genomes/species (Additional file 7). All nucleotide CDSs predicted by Prodigal were then clustered with usearch [72] and vsearch [75] at various ID thresholds (0.9, 0.8, 0.7, 0.6, 0.5). The pan-genome size at each threshold below 0.9 was then divided by the pan-genome size at 0.9 to identify the amount of pan-genome collapse due to reduced identities. The results demonstrate that most of the species showed limited pan-genome collapse at decreasing cluster thresholds (Fig. 3b). This suggests that genes in these pan-genomes are largely derived from outside of the species and do not cluster together even when the clustering threshold is low. However, *O. tsutsugamushi* demonstrated the fourth largest amount of pan-genome collapse of all species in GenBank, suggesting that the pan-genome curves at 0.9 are driven largely by divergent but homologous genes. While this metric of pan-genome collapse is largely an intrinsic measure of genomic duplication and divergence, the extent to which this happens across strains will be more accurately determined for species with representative genomic sampling. Also, despite using a similar clustering approach, slight variability was observed between clustering methods (usearch and vsearch) with regards to species demonstrating the largest amount of pan-genome collapse. In both cases, however, *Helicobacter pylori* demonstrated a greater degree of pan-genome collapse than *O. tsutsugamushi* (Additional file 8). For these reasons, the accuracy of estimations of pan-genome collapse for the species analysed here may be variable; however, our comparative pan-genome approach across 253 species is primarily intended to demonstrate the rarity of this phenomenon across bacteria.

## Discussion

In this study, we sequenced a collection of 33 *O. tsutsugamushi* strains for a genomic comparison of 40 genomes from dispersed geographical locations with a focus on Laos (*n*=5) and Thailand (*n*=13). We explored population genetics and the contributions of LGT, gene duplication and gene divergence to the genomic diversity and evolution of *O. tsutsugamushi*. We found evidence of geographical structuring despite high levels of LGT. Using several independent approaches, we found that these high levels of recombination within *O. tsutsugamushi* have eliminated most traces of a clonal frame. Repeated sequences are particularly subject to gene decay, and it is the divergence of genes and extensive intra-specific LGT that appear to be the major drivers of diversity in the *O. tsutsugamushi* pan-genome. Through pan-genome analyses, we have illustrated the great extent to which duplication and divergence drives pan-genome evolution. While the pan-genome of many other bacteria, such as *Burkholderia pseudomallei*, is driven by the acquisition of exogenous DNA [80], the adaptive potential of *O. tsutsugamushi* is driven primarily by the manipulation of endogenous DNA.

Pan-genome analyses provide insights into the functional and adaptive ability of a species or population. As we are currently severely limited in our ability to assess functional diversity of genomes, pan-genome analyses have been relegated to measuring genetic diversity. When the genetic diversity of a group (usually a species) can be completely captured by sampling all available genomes, the pan-genome is characterized as ‘closed’. However, if continued large-scale sampling of genomes is predicted to yield new gene content, the pan-genome is characterized as ‘open’ [81, 82], although the thresholds for defining novel gene content are not standardized. While small- and large-scale mutations to existent genetic material will add to the genetic diversity, a change in gene content is more likely to arise after incorporation of foreign genetic material through transduction, transformation and conjugation. The identification of novel, exogenous gene content will not be sensitive to clustering identity thresholds. Conversely, divergent genes will appear ‘identical’ at low thresholds and ‘different’ at higher thresholds. At a threshold of 0.9, the pan-genome of *O. tsutsugamushi* appears open, with an apparent increase in genetic material with additional sequencing (Fig. 3a). However, at a cluster ID of 0.5, the accumulation curve plateaus, indicating that the pan-genome is indeed closed. For species where the pan-genome is open, such as *Burkholderia pseudomallei*, lowering the cluster threshold does not change the resulting interpretation of an open pan-genome (Fig. 3a). Applying different thresholds can indicate the extent to which different evolutionary mechanisms drive the observed genetic diversity and adaptive potential of the organism.

The pan-genomes of obligate intracellular pathogens are typically characterized as closed [83] and some may have lost the ability or opportunities to gain exogenous gene content. Duplicated genes are initially genetically redundant and at risk for elimination, but maintaining diverging paralogues provide an alternative mechanism for accumulating genetic diversity and producing novel functions. For *O. tsutsugamushi*, this mechanism may be an adaptive response to the host-switching lifestyle of the species [11]. Many divergent genes that drive the pan-genome up at high cluster thresholds were annotated as versions of the gene encoding 56 kDa TSA [22]. Aside from the TSA genes, other genes were identified as conjugative secretion system-related genes, outer-membrane protein-encoding genes and mobile elements. Functions of these genes all relate either directly or indirectly to mechanisms involved in evasion of the host immune response. Therefore, host switching and evasion of host immune systems has led to diversifying selection. Gene duplication and subsequent divergence, rather than the uptake of foreign DNA through LGT, drives the *O. tsutsugamushi* pan-genome.

The phylogenetic diversity of *O. tsutsugamushi* genomes was partially illustrated through an MP tree inferred from nearly 60 000 SNPs. Low CI and RI values for the tree are indicative of high levels of homoplasy, most likely due to frequent and extensive LGT, a significant non-clonal mode of inheritance. High bootstrap support on deep nodes and topological similarities across phylogenetic methods suggest that these nodes are more robust, perhaps due to less frequent recombination across clades than within clades. Parsimony trees inferred for individual groups display high levels of homoplasy within the two main clades, suggesting that LGT is common among clade members. Conversely, the relatively high consistency among taxa at the base of the tree (encompassing multiple clades), suggests less LGT across clades. The main evolutionary trends shown in the DensiTree depiction of beast trees are highly similar to the MP tree, suggesting that while a single tree cannot capture the multiple evolutionary paths of recombined portions of the genome, the overarching evolutionary relationships among these taxa appear to be robust. This has been demonstrated previously with empirical data [30] and a simulation [79]. Homologous LGT provides an important mechanism for rapid evolutionary adaptation and phylogenetic origins that can have important implications on the diversity and evolutionary consequences of recombined regions.

Previous sub-genomic studies have reported high levels of LGT, and our mapping of HD and SD, as well as ClonalFrameML analyses, suggest that all regions of the core genome are affected. As expected, regions of high HD coincide with regions of high SD, indicating regions that have likely been recombined mostly with genomes within the sampled phylogenetic diversity. The lack of extensive regions with high SD and low HD suggest the presence of recombination from genomes outside the current dataset or rapidly evolving regions of the genome is relatively rare. Geographical barriers to recombination could, in part, explain higher levels of recombination within clades, rather than across them. However, the results of ClonalFrameML must be interpreted with caution, as the program is designed to primarily detect recombination events from sources external to the sampled population.

The high levels of LGT in the core *O. tsutsugamushi* genome have all but eliminated long, clonal regions within the genome. Methods to identify clonal regions were highly ineffective as concatenating putatively non-recombinant regions results in a parsimony trees with low CI and RI values, suggesting that these regions produce conflicting evolutionary signals and, therefore, cannot all be non-recombinant. However, when we identified the largest consecutive block from these putatively non-recombinant genomic regions (a mere 725 bp in length) and inferred a parsimony tree, the CI and RI was close to 1.0, suggesting that this region, and possibly some other regions, represent the best evidence for a clonal frame of *O. tsutsugamushi*, but cover a very small portion of the core genome (<0.01 %).

In analysing an organism with a high density of duplicated genes and high levels of recombination and LGT, we have demonstrated the utility, challenges and limitations of existing comparative genomic tools. The reduction of the *O. tsutsugamushi* genome assembled with short-read sequencing demonstrates the limitations of this approach and suggests that long-read sequencing approaches would be more appropriate in characterizing the complete genome from this highly repetitive species. We have previously demonstrated that this genome reduction can result in the appearance of missing coding regions [17], which can complicate comparative pan-genomic analyses. For phylogenetics, the high level of homoplasy complicates inferring all relationships, and demonstrates the importance of quantifying the extent and location of homoplasy before making conclusions on evolutionary relationships. We also demonstrate that running an alignment through popular methods to remove recombination is not always effective in identifying the clonal frame and the results must be evaluated to determine the effects of removing putative recombination on the underlying phylogeny. A one-size-fits-all comparative genomics approach is not always appropriate for highly recombinant, highly repetitive genomes, but combining existing tools does provide important insights into the evolution of a species.

In summary, we used whole-genome analyses to investigate evolutionary forces that might drive selection, evasion of the immune system and vaccine suitability. Investigations into the extent of recombination expand previous research and demonstrate that recombination is extensive and distributed throughout global *O. tsutsugamushi* genomes. Homologous recombination and LGT are likely facilitated by extensive repetitive sequences in the *O. tsutsugamushi* genome. Despite high levels of LGT, the pan-genome diversity across the *O. tsutsugamushi* population appears to be driven not by the acquisition of foreign DNA, but rather by a process of gene duplication and divergence to a nearly unprecedented extent compared to other bacterial species. By expanding our understanding of a globally important pathogen with high levels of repetitive DNA, duplicated genes, LGT and gene divergence, we expand concepts in bacterial evolution and microbial genomics, but also highlight challenges and limitations of current comparative genomic tools.

## Data bibliography

1. . Fleshman A, Mullins K, Sahl J, Hepp C, Nieto N *et al*. NCBI BioProject PRJNA316643, https://www.ncbi.nlm.nih.gov/bioproject/?term=PRJNA316643 (2016).
2. . Fleshman A, Mullins K, Sahl J, Hepp C, Nieto N *et al*. Figshare, https://doi.org/10.6084/m9.figshare.5995456.v1 (2018).

## Supplementary Data

Supplementary File 1
