# Corrigendum: Comparative pan-genomic analyses of *Orientia tsutsugamushi* reveal an exceptional model of bacterial evolution driving genomic diversity

**DOI:** 10.1099/mgen.0.000230

**Published:** 2018-11-01

**Authors:** Amy Fleshman, Kristin Mullins, Jason Sahl, Crystal Hepp, Nathan Nieto, Kristin Wiggins, Heidie Hornstra, Daryl Kelly, Teik-Chye Chan, Rattanaphone Phetsouvanh, Sabine Dittrich, Phonepasith Panyanivong, Daniel Paris, Paul Newton, Allen Richards, Talima Pearson

**Affiliations:** ^1^​ Northern Arizona University, Flagstaff, AZ, USA; ^2^​ Naval Medical Research Center, Silver Spring, MD, USA; ^3^​ The Ohio State University, Columbus, OH, USA; ^4^​ Lao-Oxford-Mahosot Hospital-Wellcome Trust, Research Unit, Mahosot Hospital, Vientiane, Lao People's Democratic Republic; ^5^​ Centre for Tropical Medicine and Global Health, University of Oxford, Oxford, UK; ^6^​ Lao-Oxford-Mahosot Hospital-Wellcome Trust Research Unit, Mahosot Hospital, Vientiane, Lao People's Democratic Republic; ^7^​ Foundation of Innovative New Diagnostics, Geneva, Switzerland; ^8^​ Mahidol-Oxford Tropical Medicine Research Unit, Bangkok, Thailand; ^9^​ Swiss Tropical and Public Health Institute, Basel, Switzerland; ^10^​ University of Basel, Basel, Switzerland; ^11^​ Uniformed Services University of the Health Sciences, Bethesda, MD, USA

**Keywords:** gene divergence, genome adaptation, lateral gene transfer, adaptive evolution, scrub typhus, pan-genome

There was an error in the text and files of the published article.

In Fig. 1 and the additional files, strain 18-032404 is incorrectly labeled as 18-032604.

In the additional files, strain headers for 18-032643 should be 18-030643.

In the text, the second occurrence of ‘Additional file 8’ should refer to ‘Additional file 13’.

The authors apologize for any inconvenience caused.

