# Supplementary material for: Comparative pan-genomic analyses of Orientia tsutsugamushi reveal an exceptional model of bacterial evolution driving genomic diversity
Source: Microb Genom. 2018 Jul 23;4(9):e000199. doi: 10.1099/mgen.0.000199 (PMC6202447; doi:10.1099/mgen.0.000199)
Supplement: Supplementary File 1 [file mgen-4-199-s001.pdf]

# Additional file: Metadata for isolates, DNA extraction, and bioinformatics methods.

| | ID | Country of Isolation | Province/State/Prefecture of Isolation | Region of Isolation | Date of isolation | Source of Isolation | BioSample Accession | SRA Run Accession | References |
|---|---|---|---|---|---|---|---|---|---|
| 1 | Karp (2013) | Papua New Guinea | Oro | Buna-Guna Area | 1943 | Human | SAMN04917380 | SRR3503732 | (1, 2) |
| 2 | TM2259 | Laos | Vientiane City | Xaythany District | 2008 | Human | SAMN04917381 | SRR3503734 | This Study |
| 3 | TM2325 | Laos | Vientiane City | Sikhottabong District | 2008 | Human | SAMN04917382 | SRR3503738 | (3) |
| 4 | TM2978 | Laos | Vientiane Province | Feuang District | 2009 | Human | SAMN04917383 | SRR3503739 | (3) |
| 5 | 772 | Laos | Salavan | Lao Ngam District | 2011 | Human | SAMN04917384 | SRR3503740 | This Study |
| 6 | 1768 | Laos | Luang Namtha | Namtha District | 2010 | Human | SAMN04917385 | SRR3503824 | This Study |
| 7 | Gilliam | Burma | Kachin/Sagaing | Assam Burma Border | 1944 | Human | SAMN04917386 | SRR3503829 | (4, 5) |
| 8 | TA716 | Thailand | Ubon Rachathani | Chong MekKhorat Plateau | 1963 | *Menetes berdmorei* | SAMN04917387 | SRR3503839 | (6) |
| 9 | TA763 | Thailand | Ubon Rachathani | Chong Mek, Khorat Plateau | 1963 | *Rattus rajah* | SAMN04917388 | SRR3503840 | (6) |
| 10 | Domrow | Australia | Queensland | North Queensland | 1952-54 | Human | SAMN04917389 | SRR3503847 | (7) |
| 11 | AFC-27 | Thailand | Chiang Rai | - | 1992 | Human | SAMN04917390 | SRR3503849 | (8)* |
| 12 | AFC-30 | Thailand | Chiang Rai | - | 1992 | Human | SAMN04917391 | SRR3503851 | (8)* |
| 13 | Garton | Australia | Queensland | North Queensland | 1952-54 | Human | SAMN04917392 | SRR3503852 | (7) |
| 14 | TH1811 | Thailand | Nakhon Ratchasima | Pak Tong Chai, Khorat Plateau | 1965 | Human | SAMN04917393 | SRR3503853 | (6, 9) |
| 15 | TH1812 | Thailand | Nakhon Ratchasima | Pak Tong Chai, Khorat Plateau | 1965 | Human | SAMN04917394 | SRR3503856 | (6, 9) |
| 16 | TH1814 | Thailand | Nakhon Ratchasima | Pak Tong Chai, Khorat Plateau | 1965 | Human | SAMN04917395 | SRR3503857 | (6, 9) |
| 17 | TH1817 | Thailand | Nakhon Ratchasima | Pak Tong Chai, Khorat Plateau | 1965 | Human | SAMN04917396 | SRR3503859 | (6, 9) |
| 18 | TH1826 | Thailand | Nakhon Ratchasima | Pak Tong Chai, Khorat Plateau | 1965 | Human | SAMN04917397 | SRR3503882 | (6, 9) |
| 19 | 18-032113 | Pakistan | Pakhtankhwa Khyber | Naran, Kagan Valley | 1972 | Chigger | SAMN04917398 | SRR3503883 | (10)* |
| 20 | 18-032460 | Malaysia | Perak | Ulu Kinta | 1983 | Human | SAMN04917399 | SRR3503884 | (11)* |
| 21 | 18-032404 | Malaysia | Perak | Ulu Kinta | 1983 | Human | SAMN04917400 | SRR3503885 | (11)* |
| 22 | 18-030643 | China | Fujian | Western | Early 1960s | Rodent | SAMN04917401 | SRR3503886 | (12)* |
| 23 | AFC-3 | Thailand | Chiang Rai | - | 1991 | Human | SAMN04917402 | SRR3503887 | (8)* |
| 24 | AFPL-12 | Thailand | Phitsanulok | - | 1989 | Human | SAMN04917403 | SRR3503888 | (13) |
| 25 | AFSC-7 | Thailand | Kanchanaburi | - | 1990 | Human | SAMN04917404 | SRR3503889 | (14, 15)* |
| 26 | Brown | Australia | Queensland | North Queensland | 1952-54 | Human | SAMN04917405 | SRR3503890 | (7) |

| | | | | | | | | | |
|---|---|---|---|---|---|---|---|---|---|
| 27 | BSE-125 | Solomon Islands | Temotu | Ndendo/Santa Cruz Islands | 1981 | Chigger | SAMN04917406 | SRR3503891 | (16)* |
| 28 | Citrano | Australia | Queensland | North Queensland | 1952-54 | Human | SAMN04917407 | SRR3503892 | (7) |
| 29 | Kato | Japan | Niigata Prefecture | - | 1952 | Human | SAMN04917408 | SRR3503893 | (17)* |
| 30 | MAK-119 | Taiwan | Penghu | Pescadores Islands | 1976 | Human | SAMN04917410 | SRR3503895 | (18, 19)* |
| 31 | MAK-243 | Taiwan | Penghu | Pescadores Islands | 1977 | Human | SAMN04917411 | SRR3503896 | (18, 19)* |
| 32 | Karp_ENGEN | New Guinea | Oro | Buna-Guna Area | 1943 | Human | SAMN04917412 | SRR3503897 | (1, 2) |
| 33 | Karp_NCBI | New Guinea | Oro | Buna-Guna Area | 1943 | Human | SAMN02692923 | SRS579211 | (1, 2) |
| 34 | Boryong_NCBI | South Korea | South Chungcheong | Boryong | 1989 | Human | SAMEA3138266 | ERS610201 | (20, 21) |
| 35 | Ikeda_NCBI | Japan | Niigata Prefecture | Banks of the Shinano near Takamachi | 1979 | Human | | | (22) |
| 36 | Kato_PP_NCBI | Japan | Niigata Prefecture | - | 1952 | Human | SAMN02692924 | SRS579213 | (17)* |
| 37 | TA716_NCBI | Thailand | Ubon Rachathani | Chong MekKhorat Plateau | 1963 | *Menetes berdmorei* | SAMN02693795 | SRS579607 | (6) |
| 38 | TA763_NCBI | Thailand | Ubon Rachathani | Chong MekKhorat Plateau | 1963 | *Rattus rajah* | SAMN02666744 | SRS579604 | (6) |
| 39 | UT76_NCBI | Thailand | Udon Thani | Muang District | 2003 | Human | SAMN02699164 | SRS580496 | (23) |
| 40 | Kostival | New Guinea | Oro | Dobadura Area | 1943 | Human | | | (1) |

* Daryl J. Kelly unpublished information based on personal correspondence files and inventory logs

References


1. RIGHTS FL, SMADEL JE. Studies on scrib typhus; tsutsugamushi disease; heterogenicity of strains of R. tsutsugamushi as demonstrated by cross-vaccination studies. J Exp Med. 1948 Apr 1;87(4):339-51.

2. DERRICK EH, BROWN HE. Isolation of the Karp strain of rickettsia tsutsugamushi. Lancet. 1949 Jul 23;2(6569):150.

3. Phetsouvanh R, Sonthayanon P, Pukrittayakamee S, Paris DH, Newton PN, Feil EJ, et al. The Diversity and Geographical Structure of Orientia tsutsugamushi Strains from Scrub Typhus Patients in Laos. PLOS Neglected Tropical Diseases. 2015 08/28;9(8):e0004024.

4. Bengtson IA. Apparent Serological Heterogeneity among Strains of Tsutsugamushi Disease (Scrub Typhus). Public Health Reports (1896-1970). 1945;60(50):1483-8.

5. Bennett BL, Smadel JE, Gauld RL. Studies on Scrub Typhus (Tsutsugamushi Disease). J Immunol. 1949 American Association of Immunologists;62(4):453-61.

6. Elisberg BL, Campbell JM, Bozeman FM. Antigenic diversity of rickettsia tsutsugamushi: epidemiologic and ecologic significance. J Hyg Epidemiol Microbiol Immunol. 1968;12(1):18-25.

7. CARLEY JG, DOHERTY RL, DERRICK EH, POPE JH, EMANUEL ML, ROSS CJ. The investigation of fevers in North Queensland by mouse inoculation, with particular reference to scrub typhus. Australas Ann Med. 1955 May;4(2):91-9.

8. Watt G, Chouriyagune C, Ruangweerayud R, Watcharapichat P, Phulsuksombati D, Jongsakul K, et al. Scrub typhus infections poorly responsive to antibiotics in northern Thailand. The Lancet. 1996 2017/03;348(9020):86-9.

9. Elisberg BL, Sangkasuvana V, Campbell JM, Bozeman FM, Bodhidatta P. Physiogeographic distribution of scrub typhus in Thailand. Acta Med Biol (Niigata). 1967 Dec;15:61-7.

10. Shirai A, Wisseman CL,Jr. Serologic classification of scrub typhus isolates from Pakistan. Am J Trop Med Hyg. 1975 Jan;24(1):145-53.

11. Taylor A, Sivarajah A, Kelly DJ, Lewis GE,Jr. An analysis of febrile illnesses among members of the Malaysian Police Field Force. Mil Med. 1986 Aug;151(8):442-5.

12. IMR. Annual report 1983. Institute for Medical Research, Kuala Lumpur; 1983.

13. Strickman D, Smith CD, Corcoran KD, Ngampochjana M, Watcharapichat P, Phulsuksombati D, et al. Pathology of Rickettsia tsutsugamushi infection in Bandicota savilei, a natural host in Thailand. Am J Trop Med Hyg. 1994 Oct;51(4):416-23.

14. Chao C, Garland DL, Dasch GA, Ching W. Comparative Proteomic Analysis of Antibiotic-Sensitive and Insensitive Isolates of Orientia tsutsugamushi. Ann N Y Acad Sci. 2009 05/01;1166(1):27-37.

15. Strickman D, Sheer T, Salata K, Hershey J, Dasch G, Kelly D, et al. In vitro effectiveness of azithromycin against doxycycline-resistant and -susceptible strains of Rickettsia tsutsugamushi, etiologic agent of scrub typhus. Antimicrob Agents Chemother. 1995 Nov;39(11):2406-10.

16. Shirai A, Gan E, Huxsoll D, Miles J. Serologic classification of scrub typhus isolates from Melanesia. Southeast Asian J Trop Med Public Health. 1981;12:148-50.

17. SHISHIDO A, OHTAWARA M, TATENO S, MIZUNO S, OGURA M, KITAOKA M. THE NATURE OF IMMUNITY AGAINST SCRUB TYPHUS IN MICE I. THE RESISTANCE OF MICE, SURVIVING SUBCUTANEOUS INFECTION OF SCRUB TYPHUS RICKETTSIA, TO INTRAPERITONEAL REINFECTION OF THE SAME AGENT. Jap J Med Sci Biol. 1958;11(5):383-99.

18. Olson JG, Bourgeois AL. Rickettsia tsutsugamushi infection and scrub typhus incidence among Chinese military personnel in the Pescadores Islands. Am J Epidemiol. 1977 Aug;106(2):172-5.

19. Olson JG, Bourgeois AL, Fang RCY, Coolbaugh JC, Dennis DT. Prevention of Scrub Typhus. Am J Trop Med Hyg. 1980;29(5):989-97.

20. Kim IS, Seong SY, Woo SG, Choi MS, Chang WH. High-level expression of a 56-kilodalton protein gene (bor56) of Rickettsia tsutsugamushi Boryong and its application to enzyme-linked immunosorbent assays. J Clin Microbiol. 1993 Mar;31(3):598-605.

21. Chang WH, Kang JS, Lee WK, Choi MS, Lee JH. Serological classification by monoclonal antibodies of Rickettsia tsutsugamushi isolated in Korea. J Clin Microbiol. 1990 04;28(4):685-8.

22. Tamura A, Takahashi K, Tsuruhara T, Urakami H, Miyamura S, Sekikawa H, et al. Isolation of Rickettsia tsutsugamushi antigenically different from Kato, Karp, and Gilliam strains from patients. Microbiol Immunol. 1984;28(8):873-82.

23. Blacksell SD, Luksameetanasan R, Kalambaheti T, Aukkanit N, Paris DH, McGready R, et al. Genetic typing of the 56-kDa type-specific antigen gene of contemporary Orientia tsutsugamushi isolates causing human scrub typhus at two sites in north-eastern and western Thailand. FEMS Immunology & Medical Microbiology. 2008 04/01;52(3):335-42.

## DNA Extraction

DNA was extracted by adding 1.5ml of infected L929 or Vero cells suspensions Next Advance (Averill Park, NY) tube containing the zirconium oxide beads. The tubes were vortexed and the suspension was transferred into a syringe and filtered through a 1.2 µm filter. The host cell genomic DNA was removed by adding DNaseI at a 0.01 volume and incubated at room temperature for 30 minutes. The *O. tsutsugamushi* DNA was collected by centrifugation at 11,000 g for 5 minutes. The pellet was resuspended in 300 mM of sterile sucrose. Centrifugation and resuspension in sucrose was repeated twice. 600 µl of Gentra PureGene (Qiagen, Germantown, MD) lysis buffer was added to the cells, mixed and incubated at 80ºC for 5-10 min. The lysate was then cooled and 3 µl of RNase A was added to the lysate and incubated at 37°C for 45 min. The lysate was then cooled and 200 µl of protein precipitation buffer was added. The lysate was vortexed on high setting, incubated on ice for 5 min and centrifuge at 14,000 rpm in the cooled microfuge. The supernatants were then collected and added directly to 600 µl isopropanol to precipitate the DNA (LOMWRU isolates only) or to a Phase Lock Gel tube (QuantaBio, Beverly, MA) with an equal volume of buffer-saturated phenol:chloroform (1:1) and mixed. DNA added directly to the isopropanol was allowed to precipitate at -20ºC for at least 1 hour, and centrifuged for 30 minutes at top speed in a cooled microfuge. The DNA pellets were washed twice with 70% ethanol, centrifuged for 10 minutes at top speed in a cooled microcentrifuge, and re -dissolve in TE (50 µl). The supernatants added to the Phase Lock Gel tubes were spun at top speed for 1 minute. The aqueous layer was removed and added to 600 µl isopropanol to precipitate the DNA at -20$^{O}$C overnight. Following precipitation the DNA was spun in a microcentrifuge tube for 30 min at top speed, washed twice with 70% ethanol, and spun for 15 min at top speed and re-dissolved in 20 µL TE.

## BEAST Runs and Methodologies

We chose not to incorporate tip dates because TempEst (1) revealed little, if any, correlation among time and divergence ($R^2$=0.04). We used the GTR+G model to explain the substitution processes as model selection analyses in PAUP revealed that this was the best fitting model according to the Bayesian Information Criterion. Because only variable sites were included in this analysis, we corrected for the lack of invariant sites by specifying a Constant Patterns model in the Patterns List of the BEAST xml file. We employed the Akaike's Information Criterion for MCMC samples (AICm), implemented in Tracer v1.6 (2), where we analyzed the marginal likelihood trace and found that the combination of the strict molecular clock and the constant population size demographic model fit the *O. tsutsugamushi* dataset better than combinations of the strict or uncorrelated lognormal clock models with the skygrid demographic model. Visual trace inspection and calculation of effective sample sizes was conducted

using Tracer available from http://beast.bio.ed.ac.uk/Tracer (2), confirming Markov chain Monte Carlo (MCMC) mixing within chains. However, in agreement with the low consistency indices calculated during parsimony analyses, among chain convergence did not occur, emphasizing phylogenetic uncertainty and the assortment of evolutionary histories that shapes contemporary *O. tsutsugamushi* genomes. For each dataset, four independent MCMC chains were run for 100 million generations each, with parameters and trees drawn from the posterior every 10,000$^{th}$ step. LogCombiner (3) was used to merge samples from each chain, where 20-60% of each chain was removed as burn-in to include only post-convergence samples, and all four chains were resampled every 20,000$^{th}$ step.

## References

1. Rambaut A, Lam TT, Max Carvalho L, Pybus OG. Exploring the temporal structure of heterochronous sequences using TempEst (formerly Path-O-Gen). Virus Evolution. 2016;2(1):vew007-vew.
2. Tracer v1. 6, (2014).
3. Drummond AJ, Suchard Ma, Xie D, Rambaut A. Bayesian phylogenetics with BEAUti and the BEAST 1.7. Molecular biology and evolution. 2012;29:1969-73.

Additional File: Bootstrap 50% majority rule consensus trees with 1,000 bootstrap iterations where nodes with <50% support are collapsed into polytomies. Six topologies were recovered from all sampled BEAST trees. Two of these consensus trees captured >95% of all trees and are shown below. Blue tree represents the topology of 48.74% of trees while the orange topology represents 46.64% of trees. Overlapping topology causes the semitransparent lines of each tree to appear red.

## Trees rooted with *R. bellii* for discovering root within *O. tsutsugamushi.*

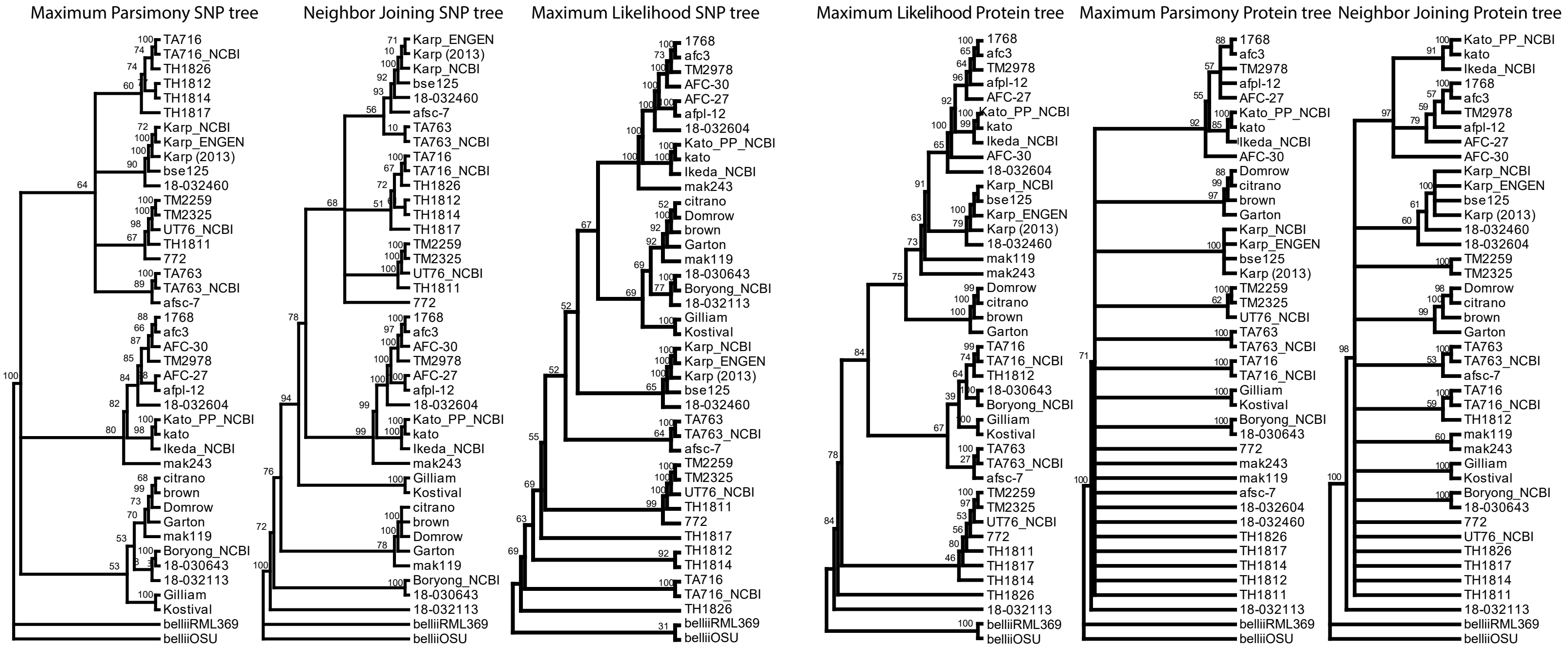


## SNP trees with only *O. tsutsugamushi.*

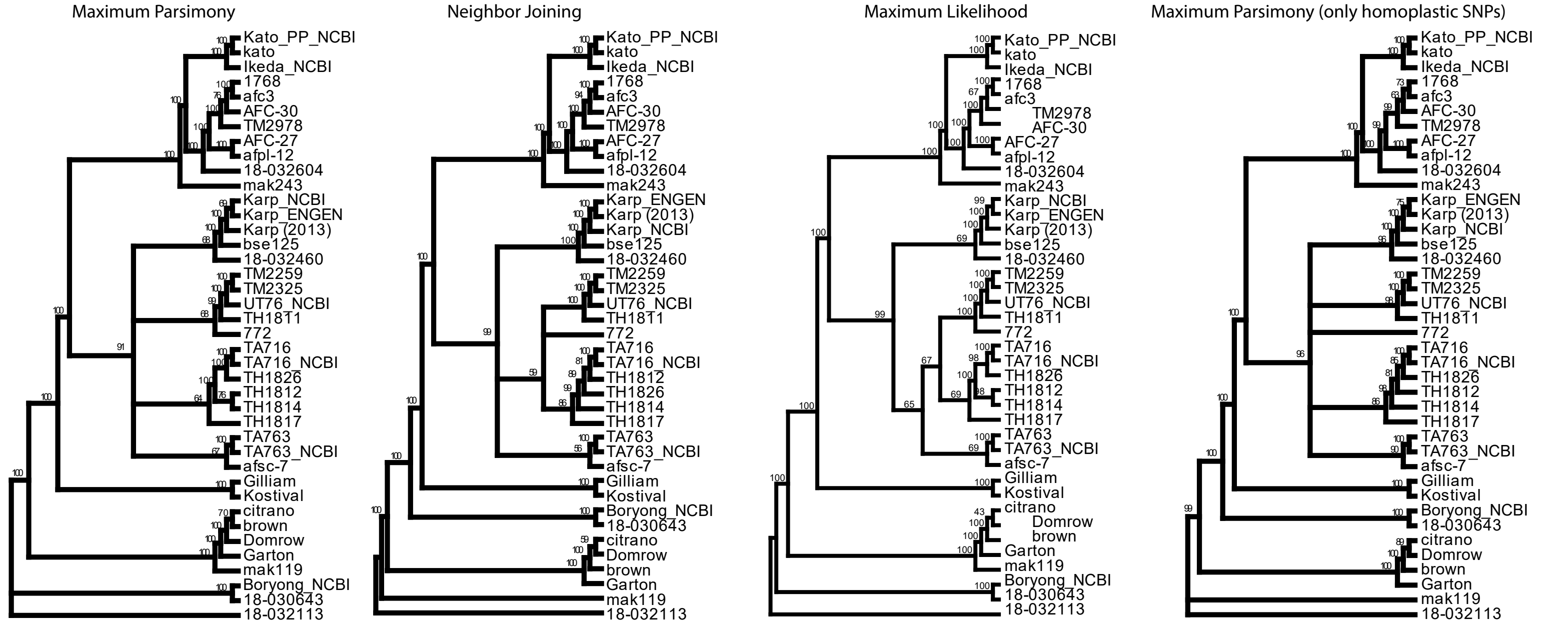


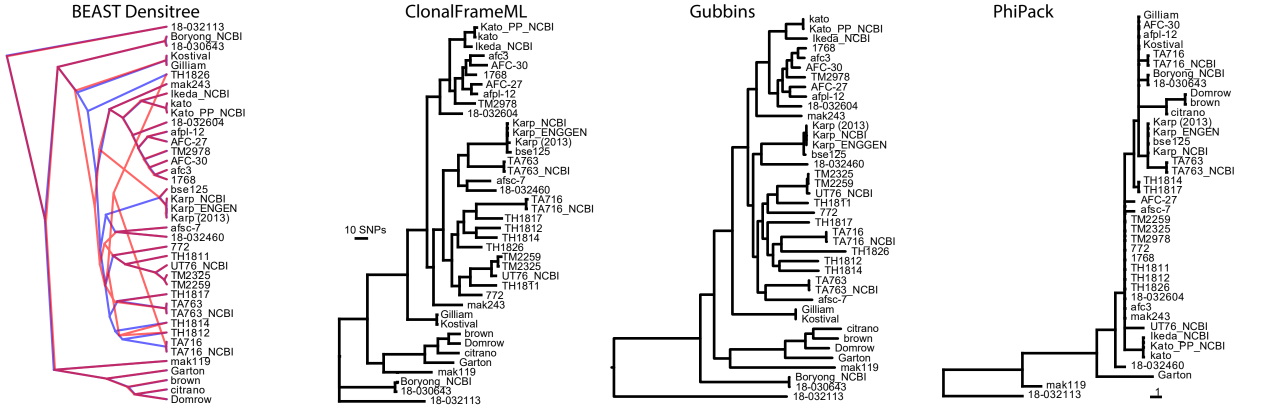

Additional file: Protein alignment demonstrating genomic divergence.

```
Ikeda    MPSVTKYCLKSSIFAMHNPSSMCKNMLYYRLNKHYSAFRIYVCGYIFLFLLQNTCKNHILCRNSLLESYSVLIFSDLYTPSHLF-YNSLCLYYFYHILLLIY--------------------------------------------------
Boryong  MPSVTRYYLKSNIFAMHNPSLIHKNMLYYRLNKYYSAFHIYV--------------------------------------------------------------------------------------------------------------
mak 119  MPSVTKYYLKSNIFAMHNPSLIHKNMLYYMMNKYYSTFHTYV--FVVSYIMQKPSTRVII--------FSSHFFLEIFILHHIFVYNSLCLYYFYHILLLIYSCNPYSKYTLTISPYIEISNSDLLNRNFLIESVFLVYLKKHLHTVLILIS
```
